# The Institutional Library

**Published:** 1913-10-04

**Authors:** 


### THE INSTITUTIONAL LIBRARY.

### The Prototype of Sherlock Holmes.

Joseph Bell, M.D., F.R.C.S., J.P., D.L.: An Appreciation. (Edinburgh and London: Oliphant, Anderson, and Ferrier. 1913. Pp. 92. Price 3s. 6d. net.)

Among the Edinburgh worthies of the generation now fast melting away few exhibited finer character, more fascinating personality than Joseph Bell, some details of whose life and career were set forth in The Hospital at the time of his death (October 14, 1911). Outside the medical and University circles of Auld Reekie, his reputation was perhaps most widely known as the prototype from which Sir A. Conan Doyle created the famous detective, Sherlock Holmes. Mrs. Jessie Saxby, the author of this brief biography, is indignant because popular error, so she says, has confused the methods and the man; and she protests that, though Conan Doyle admittedly modelled Holmes' observations and deductions on those of Dr. Bell, the latter's character was as different as possible from that of the cocaine-taking thief-catcher. To the best of our belief the confusion to which she alludes has no existence real enough to require quite so much indignation; but if it ever has had, at least the delusion will hardly survive any longer. For the rest, the authoress allows Dr. Bell to reveal himself mainly in extracts from letters, which show him to have been deeply religious, yet with a keen sense of humour; generous and charitable, yet avoiding all publicity in his well-doing; cool and well-balanced, yet affectionate and sympathetic. In short, a really good friend and a really good man; good enough, in fact, to have deserved a better biography.

Massage. By Douglas Graham, M.D. (Philadelphia and London: J. B. Lippincott Company. Fourth Edition. 1913. Pp. 574. Price 21s. net.)

The first edition of this massive and splendidly produced book was published as long ago as 1884; and we are quite prepared to believe the author's statement that it was the first text-book on the subject in the English language. Since that date massage has come more and more to the front, and is now generally recognised as quite one of the most valuable branches of physical therapeutics. Dr. Graham, too, has progressed with the times; and his book maintains its position in the front rank of the now numerous monographs on this subject. He does not quite keep himself clear of the tendency, which is so prevalent among writers on all forms of physico-therapy, to speculate rather rashly on the exact rationale of the benefits to be derived from massage. But he gives a thorough and complete account of the science and practice of massage, from Homeric times to 1913, which must prove of the greatest value to all who study it.

How to Take Care of the Sick at Home. By W. and S. Rintoul.

How to Take Care of Baby. By Selina F. Fox, M.D. (London: Women's Industrial Council. Price 1d. each.)

These pamphlets are designed presumably to help those whose means do not allow them to purchase more expensive works. Excellent as they are for the price charged, we cannot help thinking that in homes so humble instruction by the written word alone would hardly be of much avail. Of the two pamphlets submitted to us, we prefer Dr. Fox's, which is excellent. The other one is rather too haphazard in arrangement, but is quite sound in doctrine as far as it goes.

Medical Electricity and Light. An Elementary Text-book for Nurses. By Ettie Sayer, M.B., B.S.(Lond.). With 10 Plates and 33 Diagrams. (London: The Scientific Press, Ltd. 1913. Price 3s. 6d.)

This text-book, unlike far too many publications of recent years, has ample justification for its existence. Many a nurse engaged in the electrical departments of our hospitals must have longed for an elementary guide to assist her during the first few weeks of her struggle to become acquainted with the rationale of the intricate instruments and the complex "treatments" she has had to control. Dr. Ettie Sayer has managed her task very well on the whole, and has been ably seconded by the publishers in the matter of plates and illustrations. The chief fault to be found in the book is evidence of a want of thoroughness in revision. There are many loosely worded sentences and a few striking grammatical sins have been committed. But these will not detract from the usefulness of the book to nurses engaged in electrical work, for it would be difficult to improve on the way in which the writer unravels, step by step, the difficulties lying in the path of those nurses who are endeavouring to be something more than mere turners of switches. The theory and practice of galvanism, faradism, high frequency, radiant energy, and static electricity are admirably explained; chapters on ionic medication, radium, and *x*-rays are no less useful. Medical men who practise this specialty will also owe a debt of gratitude to Dr. Ettie Sayer, for her text-book should go far to encourage nurses and assist them in becoming proficient helpers in this branch of therapeutics, and so lessen the difficulty in finding properly trained assistants.

### Treatment After Operations.

Treatment after Operations. By Mary Wiles. (London: The Scientific Press, Ltd. Pocket Guide Series. Price 1s. net.)

This latest addition to the useful series of little handbooks issued for nurses under the name of the Pocket Guide Series is eminently practical in its tenor. Though necessarily brief, the notes on the various operation cases are very much to the point, and likely to prove of practical utility. The form of the book allows of easy reference, and its portability must recommend itself as a real *vade mecum*. Some of the statements are perhaps rather drastically dogmatic, but the author is a safe guide in general principles. We must demur to the suggestion that feathers may under any circumstances be poked into a tracheotomy tube, even if they are sterilised. This little book will undoubtedly serve a useful purpose.

Pye's Elementary Bandaging and Surgical Dressing. Revised and partly rewritten by W. H. Clayton-Greene, F.R.C.S., assisted by V. Z. Cope, M.S. (Lond.). Thirteenth Edition. (Bristol: John Wright and Sons, Ltd. 1913. Price 2s. net.)

Although this well-known little manual does not now reign unrivalled among its class as it did in the late eighties, yet it has undoubtedly maintained a high position among its numerous competitors. Founded as we all know on that splendid old text-book, Pye's "Surgical Handicraft," this pocket companion has been the friend of house surgeons and dressers for a quarter of a century. It is the dresser's guide and the casualty officer's library.

A book with such a long history is apt to show signs of the obsolete, but modern methods are plainly in evidence here, and in spite of its small size it contains a large amount of practical detail and a fair number of illustrations. A chapter on poisoning cases has been entrusted to the care of Dr. W. Wilcox, and Mr. Cope has added a good deal of modern detail to this edition. The type used is of necessity rather small and trying to the eyes owing to the desire to keep the book so small, but we feel that it would be good policy to employ larger type even at the expense of increasing the number of pages (not their size).

Ionic Medication. By H. Lewis Jones, M.D., F.R.C.P. (London: H. K. Lewis. 1913. Pp. 151. Price 5s. net.)

When ionic medication was first introduced a few years ago it had the misfortune to undergo a "boom" at the hands of some over-enthusiastic physicians, who jumped to conclusions which were unwarranted and were eventually disproved by the lapse of time. As usually happens in such circumstances, a certain amount of prejudice against the method was thus created in the minds of the more conservative type of therapeutist, and the effects of this reaction have by no means yet disappeared. Sufficient time has, however, elapsed for impartial and expert opinion to crystallise; and the potentialities, as well as the limitations, of ionic medication are in consequence much better understood. It would be hard to select anyone better qualified than Dr. Lewis Jones to sum up the fruits of recent experience of this method; and it is matter for congratulation that the want of a text-book on it has now been abolished by his capable pen. The book is not too long, yet omits nothing which it should contain; does not claim too much, yet is full of faith in ionic medication for certain conditions; in short, is a valuable guide to either a professed electro-therapeutist or a general practitioner. We recommend it with unusual confidence.

### Incipient Phthisis, and Thermal Environment.

Incipient Pulmonary Tuberculosis. By D. B. Lees, M.D., F.R.C.P. (London: H. K. Lewis. 1913. Pp. 116. Price 5s. net.)

This book contains Dr. Lees' Bradshaw Lectures of 1912. It is devoted to upholding three principal theses, two of which will probably obtain the concurrence of most experts in this particular branch of medicine. One is that incipient pulmonary tuberculosis can often be diagnosed on the physical signs, especially percussion in the recumbent position, long before any bacteriological evidence is obtainable, and at a time when auscultatory signs are still doubtful. Another is that a negative bacteriological report (for tubercle bacilli in the sputum) is often deceptive, and should not be thought to invalidate a diagnosis made upon other *data*. The third is that prompt and permanent arrest of early phthisis can always be secured by the method of continuous antiseptic inhalation, which Dr. Lees has advocated for many years. The advocacy throughout is skilful, and the whole essay well worth reading.

Studies on the Influence of Thermal Environment on the Circulation and the Body-Heat. By Edgar R. Lyth, M.B. (London: John Bale, Sons, and Danielsson, Ltd. 1913. Price 2s. 6d. net.)

This monograph contains the results of more than twenty-five thousand observations of the pulse-rate, the blood-pressure, and the superficial and deep temperatures of the body under various thermal conditions. The conclusions arrived at are not exactly revolutionary, but confirm the recognised establishment of an equilibrium of the circulatory apparatus, comprising "as regards the blood-vessels a suitable degree of muscular contraction of their coats, and as regards the heart a suitable rate and force of its pulsations. Besides determining the pressure and the speed of the current, these factors involve a corresponding distribution of the blood."

### The Pocket Guide Series.

Principal Drugs and their Uses. By A Pharmacist. (London: The Scientific Press. 1913. Pp. 95. Price 1s. net.)

The Nurse's Duties Before and During Operations. By E. Margaret Fox, Matron of the Prince of Wales's Hospital, Tottenham. (London: The Scientific Press. 1913. Pp. 110. Price 1s. net.)

Two more volumes of this admirable series have now been published, and entirely confirm the highly favourable impressions which we based upon the first members of the collection. "Principal Drugs and their Uses" is intended for the use of nurses, who must often experience a healthy and not unnatural curiosity about drugs which they are constantly administering to patients. The author starts off by dealing with poisons and dangerous drugs. After mentioning the substances which are included in the Schedules of Poisons attached to the Pharmacy Acts, he points out that these lists are incomplete, and proceeds to a lengthy list of dangerous drugs, of which some are in the Poisons Schedules, and others are not. After a few pages of useful definitions showing what the descriptive terms mean which are applied to drugs, he deals in turn with drugs in most common use, giving their popular, as well as their scientific names, the nature of each, and a brief account of the dosage and uses of the various preparations mentioned above. One or two omissions may, however, be mentioned and suggested for inclusion in the next edition. Stovaine, eucaine, and novocain are not mentioned, whereas cocaine receives a good deal of the attention they might well claim to share in. Aspirin is described as the trade name of a synthetic compound; but neither the true name nor the dose is mentioned. On the other hand, adrenalin (which is every bit as much a trade name as aspirin) is given without comment; and suprarenal extract does not appear in the list at all. Chloretone and Warburg's tincture are not included. Finally, a valuable section on poisons and antidotes concludes the book, which should achieve rapid popularity among those for whom it is intended.

Miss Fox's little book is of an even higher order of merit. Indeed, we incline to the opinion that it is the best of the Pocket Guide Series so far, which is saying a great deal. The whole tone of her teaching is so sensible, so thoroughly sound, both in technical and non-technical details, so free from gush, that we should be tempted to describe it as "manly" if we were not afraid of Miss Fox's discontent with such an adjective. The language used is perfectly frank and clear, yet the style is by no means bald or dull; indeed, we know of few women authors who can go so straight to the point in accurate and simple English prose. Nothing is omitted, and nothing superfluous is put in; in short, the whole essay is most refreshing—a pleasure to read through, and full of superb common sense.

